# Long-term follow-up of dynamic brain changes in patients recovered from COVID-19 without neurological manifestations

**DOI:** 10.1172/jci.insight.155827

**Published:** 2022-02-22

**Authors:** Tian Tian, Jinfeng Wu, Tao Chen, Jia Li, Su Yan, Yiran Zhou, Xiaolong Peng, Yuanhao Li, Ning Zheng, Aoling Cai, Qin Ning, Hongbing Xiang, Fuqiang Xu, Yuanyuan Qin, Wenzhen Zhu, Jie Wang

**Affiliations:** 1 Department of Radiology, Tongji Hospital, Tongji Medical College, Huazhong University of Science and Technology, Wuhan, Hubei, China.; 2 Key Laboratory of Magnetic Resonance in Biological Systems, State Key Laboratory of Magnetic Resonance and Atomic and Molecular Physics, National Center for Magnetic Resonance in Wuhan, Wuhan Institute of Physics and Mathematics, Innovation Academy for Precision Measurement Science and Technology, Chinese Academy of Sciences-Wuhan National Laboratory for Optoelectronics, Wuhan, Hubei, China.; 3 Institute and Department of Infectious Disease and; 4 Department of Anesthesiology, Tongji Hospital, Tongji Medical College, Huazhong University of Science and Technology, Wuhan, Hubei, China.; 5 University of Chinese Academy of Sciences, Beijing, China.

**Keywords:** COVID-19, Neuroscience, Neuroimaging

## Abstract

**BACKGROUND:**

After the initial surge in COVID-19 cases, large numbers of patients were discharged from a hospital without assessment of recovery. Now, an increasing number of patients report postacute neurological sequelae, known as “long COVID” — even those without specific neurological manifestations in the acute phase.

**METHODS:**

Dynamic brain changes are crucial for a better understanding and early prevention of “long COVID.” Here, we explored the cross-sectional and longitudinal consequences of COVID-19 on the brain in 34 discharged patients without neurological manifestations. Gray matter morphology, cerebral blood flow (CBF), and volumes of white matter tracts were investigated using advanced magnetic resonance imaging techniques to explore dynamic brain changes from 3 to 10 months after discharge.

**RESULTS:**

Overall, the differences of cortical thickness were dynamic and finally returned to the baseline. For cortical CBF, hypoperfusion in severe cases observed at 3 months tended to recover at 10 months. Subcortical nuclei and white matter differences between groups and within subjects showed various trends, including recoverable and long-term unrecovered differences. After a 10-month recovery period, a reduced volume of nuclei in severe cases was still more extensive and profound than that in mild cases.

**CONCLUSION:**

Our study provides objective neuroimaging evidence for the coexistence of recoverable and long-term unrecovered changes in 10-month effects of COVID-19 on the brain. The remaining potential abnormalities still deserve public attention, which is critically important for a better understanding of “long COVID” and early clinical guidance toward complete recovery.

**FUNDING:**

National Natural Science Foundation of China.

## Introduction

The ongoing process of responding to and recovering from COVID-19 has been a global focus since its onset and has brought an unprecedented challenge to public health (1, 2). In particular, the highly infectious SARS-CoV-2 has an organotropism beyond the respiratory tract (3), setting the stage for the development of short-term and long-term clinical sequelae in multiple organs.

SARS-CoV-2 is neurotropic (4) and has caused numerous neurological abnormalities in acute and chronic stages, such as encephalopathy, encephalitis, stroke, peripheral neuropathy, anosmia, dysgeusia, amnesia, and psychosis (3, 5–7). After the initial surge of infection, large numbers of hospitalized patients were discharged without assessment of recovery. Now, an increasing number of patients are reporting postacute neurological sequelae (8, 9) — even those without specific neurological manifestations in the acute phase; this is known as “long COVID” (10). Early prevention, recognition, and management of COVID-19–related neurological disorders are both crucial and challenging. Moreover, it is equally important and meaningful for patients without clear neurological manifestations (11), since they account for a large proportion of the pandemic; however, their increased risk of suffering from “long COVID” (5, 12) has been more easily ignored. Particularly, scientists should investigate and explore the pathophysiology of “long COVID” to ensure that discharged patients receive early diagnosis and timely appropriate treatments, especially for the elderly patients who are associated with higher comorbidity and hospitalization rates and poorer prognoses (13).

So far, the potential pathogenesis of “long COVID” remains unclear. A comprehensive snapshot of the neurological disorders during the acute and chronic stages of COVID-19 has demonstrated complex and long-term brain damage. Further neuroimaging studies have contributed to the differential prediction or diagnosis of COVID-19–related neurological effects and are essential for determining the underlying neuropathogenesis that will guide intervention treatments. Our previous 3-month follow-up study based on magnetic resonance imaging (MRI) emphasized the long-term changes of microstructures and cerebral blood flow (CBF) in discharged elderly patients without neurological manifestations (14). However, little is known of the longer follow-up and longitudinal consequences of COVID-19 on the brain. Longitudinal follow-up studies are required to ascertain the long-term neurological effects of the COVID-19 pandemic and shed light on related mechanisms.

We performed a second follow-up study to explore the long-term neuroimaging changes in discharged elderly patients without clear neurological manifestations based on our previous 3-month follow-up study (14). Thirty-four 50- to 70-year-old patients were reevaluated by quantitative MRI and state-of-the-art postprocessing protocols at 10 months after discharge. A clearer understanding of the brain dynamics of COVID-19 after discharge contributed to revealing the neuropathogenesis and recovery mechanisms of long-term injury in COVID-19 patients, and this may be critically important for early clinical guidance toward complete recovery.

## Results

**Figure 1:**
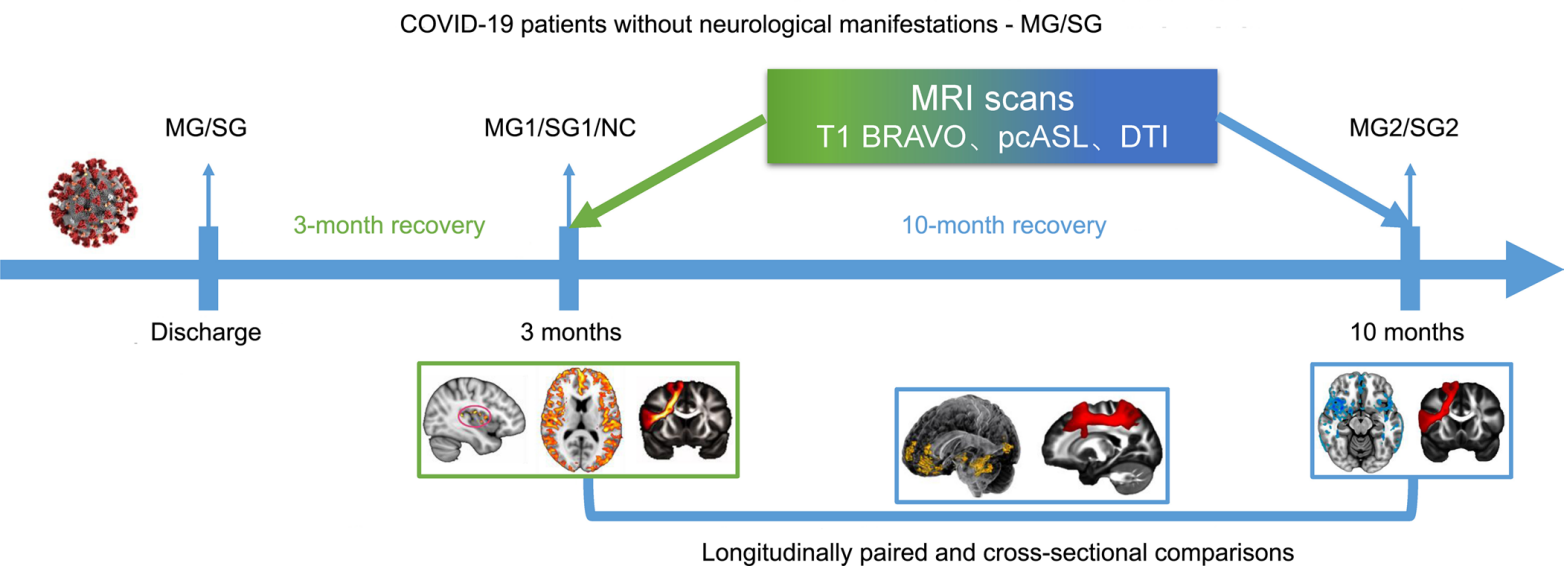
Flow diagram of the experimental design. Five data sets were acquired in follow-up studies. Green color, relative data have been published in our former work (14); blue color, research content focused in this study. BRAVO, brain volume; DTI, diffusion tensor imaging; MG1, mild group at 3 months after discharge; MG2, mild group at 10 months after discharge; NC, normal control; pcASL, pseudocontinuous arterial spin labeling; SG1, severe group at 3 months after discharge; SG2, severe group at 10 months after discharge.

A flow diagram of the experimental design is succinctly shown in Figure 1. Five data sets were acquired in follow-up studies, including: (a) normal control (NC) group; (b) mild group at 3 months after discharge (MG1); (c) mild group at 10 months after discharge (MG2); (d) severe group at 3 months after discharge (SG1); and (e) severe group at 10 months after discharge (SG2). The 3-month consequences of COVID-19 on the brain have been published in our former study (14). This study further explored: (a) paired comparisons performed within the different recovered periods of MG and SG; (b) 10-month consequences of COVID-19 on the brain of discharged patients compared with the NC group; and (c) differences in brain alterations between SG and MG at 10 months after discharge.

### Demographics and clinical characteristics.

Demographic and clinical characteristics data are listed in Table 1. The interval between the 2 time points was 202.31 ± 14.42 days (mean ± SD). The mean duration from discharge to the second follow-up was 302.70 ± 15.58 days. Thirty-one age-, sex-, and education-matched non–COVID-19 volunteers were enrolled in the NC group. There were significant differences in age, history of hypertension, and history of diabetes between some group comparisons. To compensate for the influence of these chronic diseases on the brain, it was important to include them as covariates for further analysis. For inflammatory markers, we found significant elevations in C-reactive protein (CRP), procalcitonin (PCT), IL-6, and IL-2 receptor (IL-2R) in the SG compared with the MG.

### Cortical thickness and CBF comparisons.

**Figure 2:**
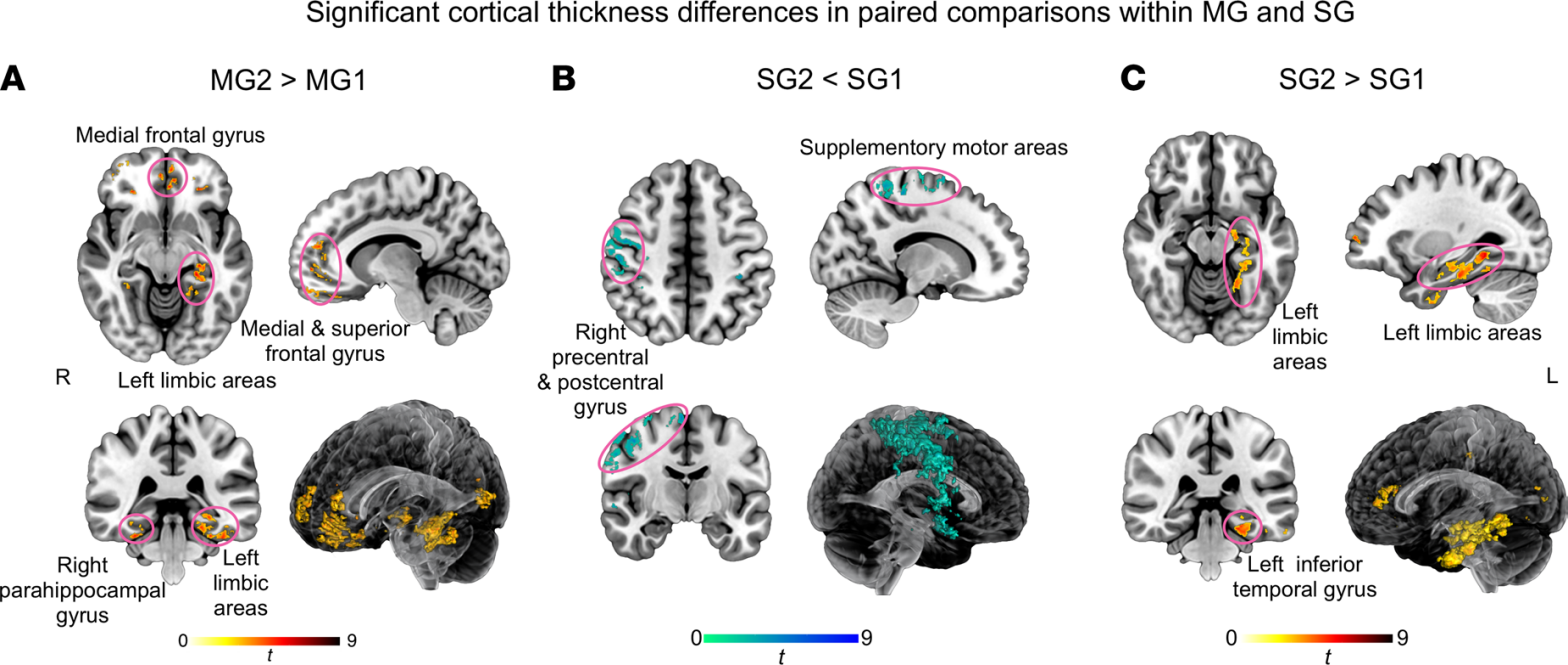
Cortical thickness analyses. (**A**–**C**) There were only significant differences in paired comparisons. L, left; MG1, mild group at 3 months after discharge; MG2, mild group at 10 months after discharge; R, right; SG1, severe group at 3 months after discharge; SG2, severe group at 10 months after discharge.

For the cortical thickness analysis, there were only significant differences in paired comparisons (threshold-free cluster enhancement [TFCE], *P* < 0.05 family-wise error corrected). Compared with MG1, MG2 exhibited thicker gray matter in the left limbic areas, right parahippocampus, bilateral frontal cortex, and left temporal-parietal cortex (Figure 2A). Meanwhile, compared with SG1, SG2 displayed not only gray matter atrophy in the right sensorimotor areas and right temporal-parietal cortex (Figure 2B), but also thicker gray matter in the left limbic areas and left temporal-frontal cortex (Figure 2C). Detailed information regarding clusters is provided in Supplemental Methods (supplemental material available online with this article; https://doi.org/10.1172/jci.insight.155827DS1). No significant differences were observed in NC-MG2, NC-SG2, and MG2-SG2 comparisons.

**Figure 3:**
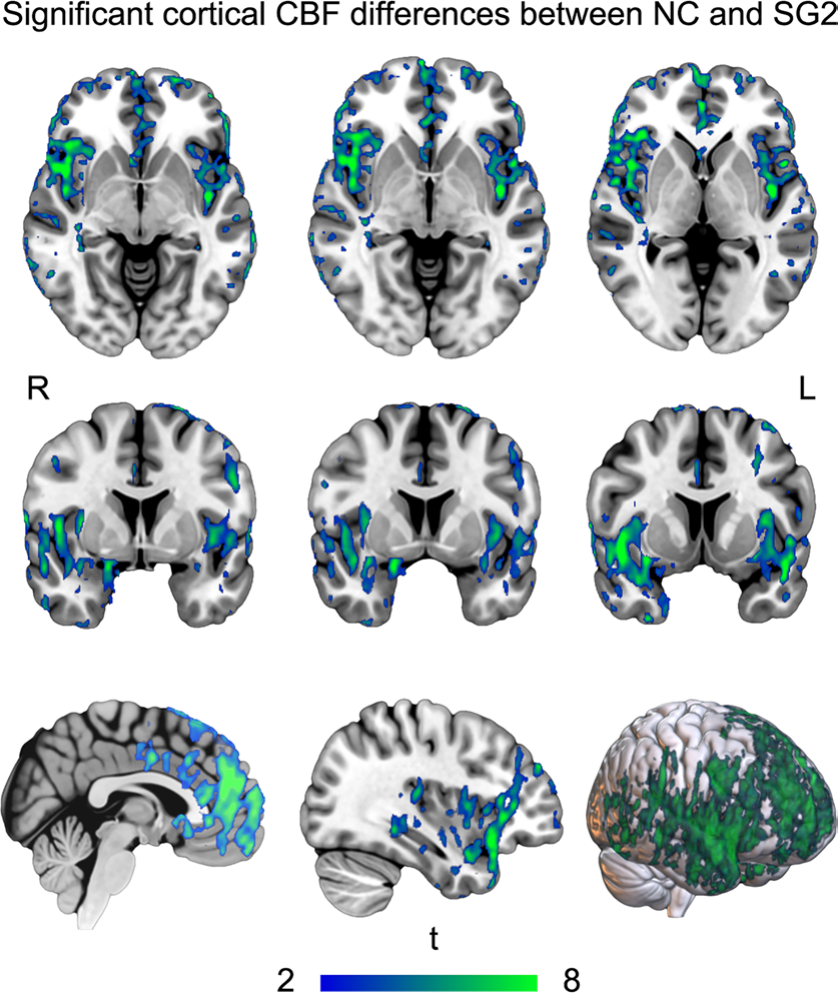
Cortical CBF analyses. Compared with NC, SG2 showed extensive lower CBF values in the brain. CBF, cerebral blood flow; L, left; NC, normal control; R, right; SG2, severe group at 10 months after discharge.

For statistical comparisons of cortical CBF, significant differences were observed between NC and SG2 (TFCE, *P* < 0.05 family-wise error corrected). SG2 showed extensive lower CBF values in the brain (Figure 3), especially in bilateral frontal cortices and temporal cortices. Compared with the previous 3-month findings (14), the cortical areas of hypoperfusion were reduced; however, the peak value was still observed in the left insula (Montreal Neurological Institute [MNI] coordinates: –43, –7, 4, NC > SG2, *t* test value = 6.28).

### Subcortical nuclei volume and CBF comparisons.

**Figure 4:**
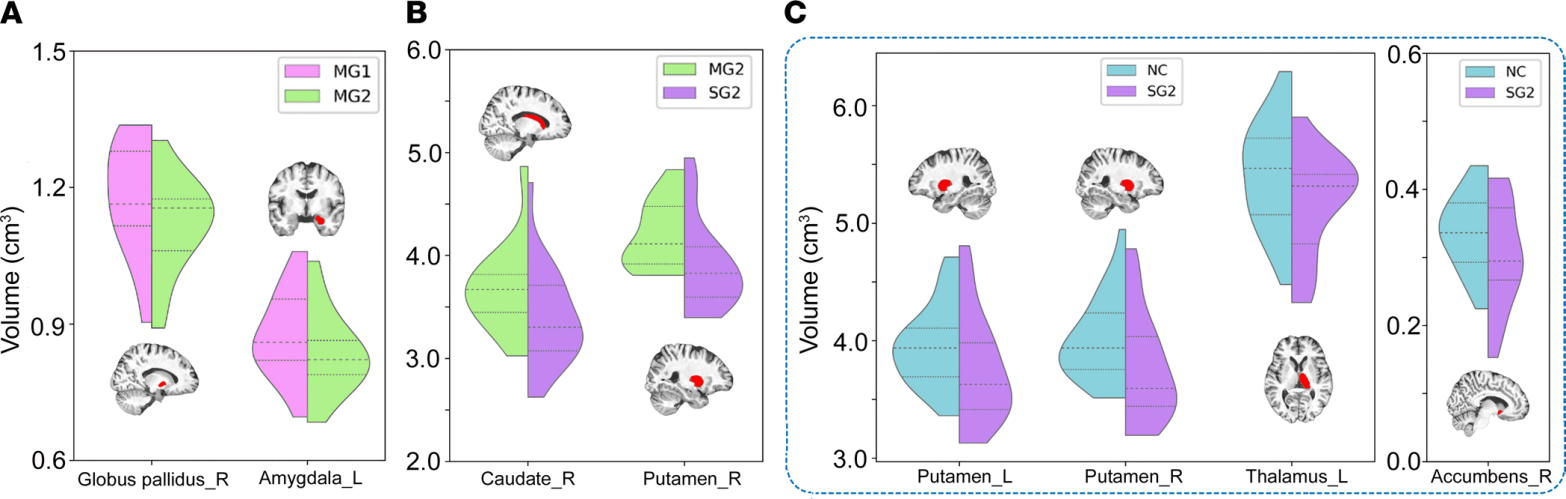
Subcortical nuclei with significant volumetric differences between groups. (**A**–**C**) Significant (*P* < 0.05) volumetric differences in subcortical nuclei were found in MG paired, SG2-MG2, and SG2-NC comparisons. L, left; MG1, mild group at 3 months after discharge; MG2, mild group at 10 months after discharge; NC, normal control; R, right; SG2, severe group at 10 months after discharge.

For the subcortical nuclei analysis, 14 nuclei were obtained from preprocessing in bilateral caudate, putamen, thalamus, globus pallidus, hippocampus, amygdala, and accumbens. After FDR correction, no surviving results were observed in subcortical nuclei volume and CBF analysis. Considering that this is an exploratory study (not a confirmatory study) with a small sample size, even if the results cannot pass the strict correction, an uncorrected threshold (*P* < 0.05) was still used to show the significance trends. The volumes of right globus pallidus (*t* = –3.00, *P* = 0.0029) and left amygdala (*t* = –2.40, *P* = 0.0186) were significantly lower in MG2 than MG1 (Figure 4A). SG2 showed significantly lower volumes in the right caudate (*t* = –1.91, *P* = 0.0325) and right putamen (*t* = –2.22, *P* = 0.0176) compared with MG2 (Figure 4B), as well as the left putamen (*t* = –1.77, *P* = 0.0409), right putamen (*t* = –2.22, *P* = 0.0152), left thalamus (*t* = –1.88, *P* = 0.0350), and right accumbens (*t* = –1.70, *P* = 0.0480) compared with NC (Figure 4C). No significant differences were observed in NC-MG2 and SG2-SG1 comparisons.

**Figure 5:**
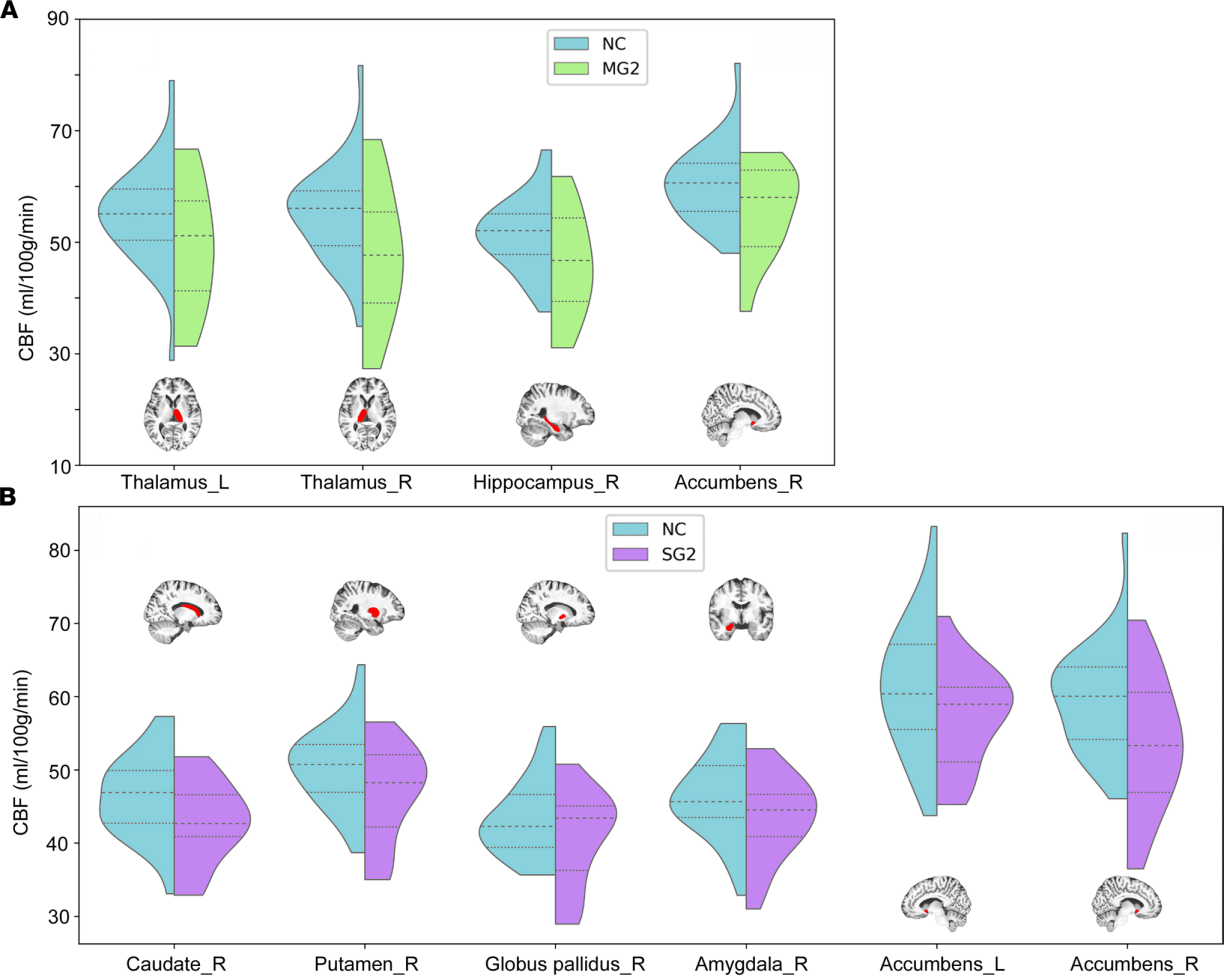
Subcortical nuclei with significant CBF differences between groups. (**A** and **B**) Both MG2 and SG2 showed significantly reduced CBF than NC in many subcortical nuclei. CBF, cerebral blood flow; L, left; MG2, mild group at 10 months after discharge; NC, normal control; R, right; SG2, severe group at 10 months after discharge.

For the subcortical nuclei CBF alterations, significant differences were only observed between NC and patients. Compared with NC, MG2 exhibited significantly reduced CBF values in the left thalamus (*t* = –1.70, *P* = 0.0487), right thalamus (*t* = –2.29, *P* = 0.0132), right hippocampus (*t* = –2.12, *P* = 0.0198), and right accumbens (*t* = –1.90, *P* = 0.0305) (Figure 5A). Compared with NC, SG2 showed significantly lower CBF values in the right caudate (*t* = –2.63, *P* = 0.0143), right putamen (*t* = –2.63, *P* = 0.0147), right globus pallidus (*t* = –1.71, *P* = 0.0482), right amygdala (*t* = –1.75, *P* = 0.0447), left accumbens (*t* = –1.83, *P* = 0.0389), and right accumbens (*t* = –2.64, *P* = 0.0073) (Figure 5B). No significant differences were observed in MG2-SG2, MG2-MG1, and SG2-SG1 comparisons.

### White matter tract analysis.

After FDR correction, no surviving results were observed in the white matter tract analysis. As mentioned above, an uncorrected threshold (*P* < 0.05) was still used to show the significance trends.

**Figure 6:**
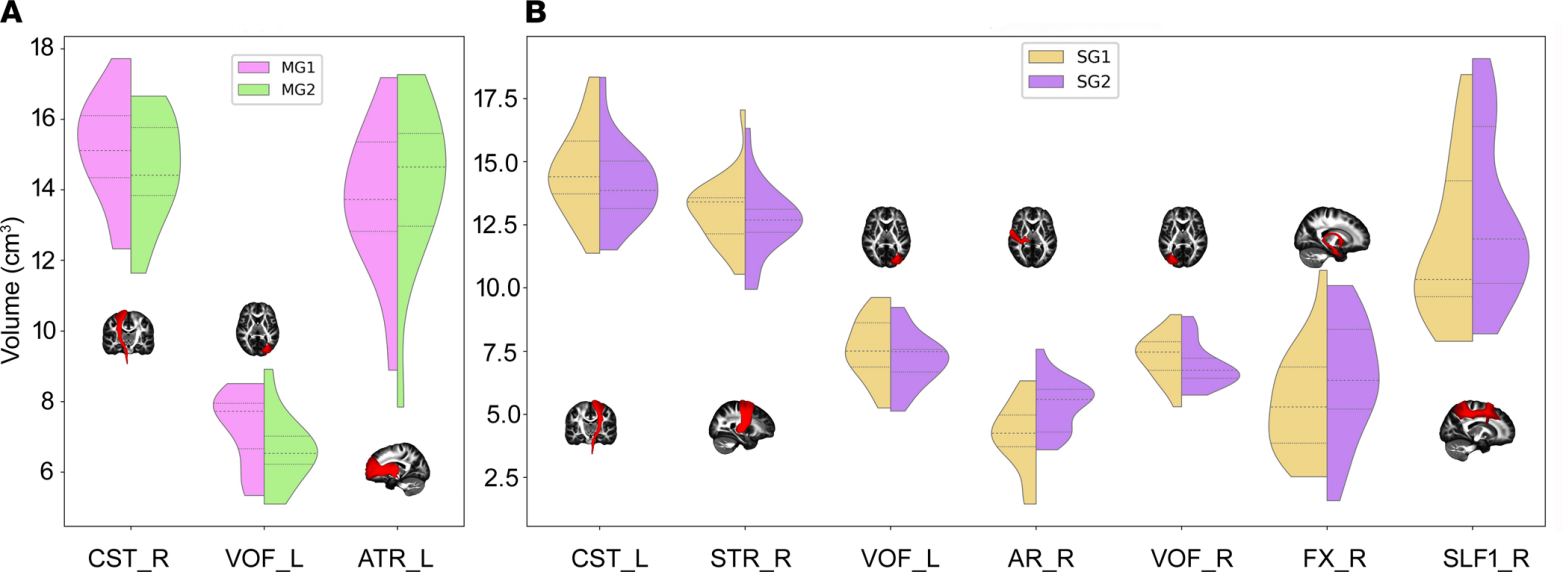
White matter tracts with significant volumetric differences in paired comparisons. (**A** and **B**) There were significant volumetric differences in tracts between the different recovered periods of MG and SG. AR, acoustic radiation; ATR, anterior thalamic radiation; CST, corticospinal tract; FX, fornix; L, left; MG1, mild group at 3 months after discharge; MG2, mild group at 10 months after discharge; R, right; SG1, severe group at 3 months after discharge; SG2, severe group at 10 months after discharge; SLF1, superior longitudinal fasciculus I; STR, superior thalamic radiation; VOF, vertical occipital fasciculus.

Compared with MG1, MG2 showed a significantly greater volume in the left anterior thalamic radiation (ATR) (*t* = 2.04, *P* = 0.0274) and lower volumes in the right corticospinal tract (CST) (*t* = –1.99, *P* = 0.0354) and left vertical occipital fasciculus (VOF) (*t* = – 2.38, *P* = 0.0178) (Figure 6A). Compared with SG1, SG2 exhibited significantly greater volumes in the right acoustic radiation (AR) (*t* = 5.34, *P* = 0.0002), right fornix (FX) (*t* = 2.15, *P* = 0.0256), and right superior longitudinal fasciculus I (SLF1) (*t* = 2.20, *P* = 0.0164), as well as lower volumes in the left CST (*t* = –2.16, *P* = 0.0252), right superior thalamic radiation (STR) (*t* = –2.15, *P* = 0.0258), left VOF (*t* = – 2.56, *P* = 0.0098), and right VOF (*t* = – 1.84, *P* = 0.0440) (Figure 6B).

**Figure 7:**
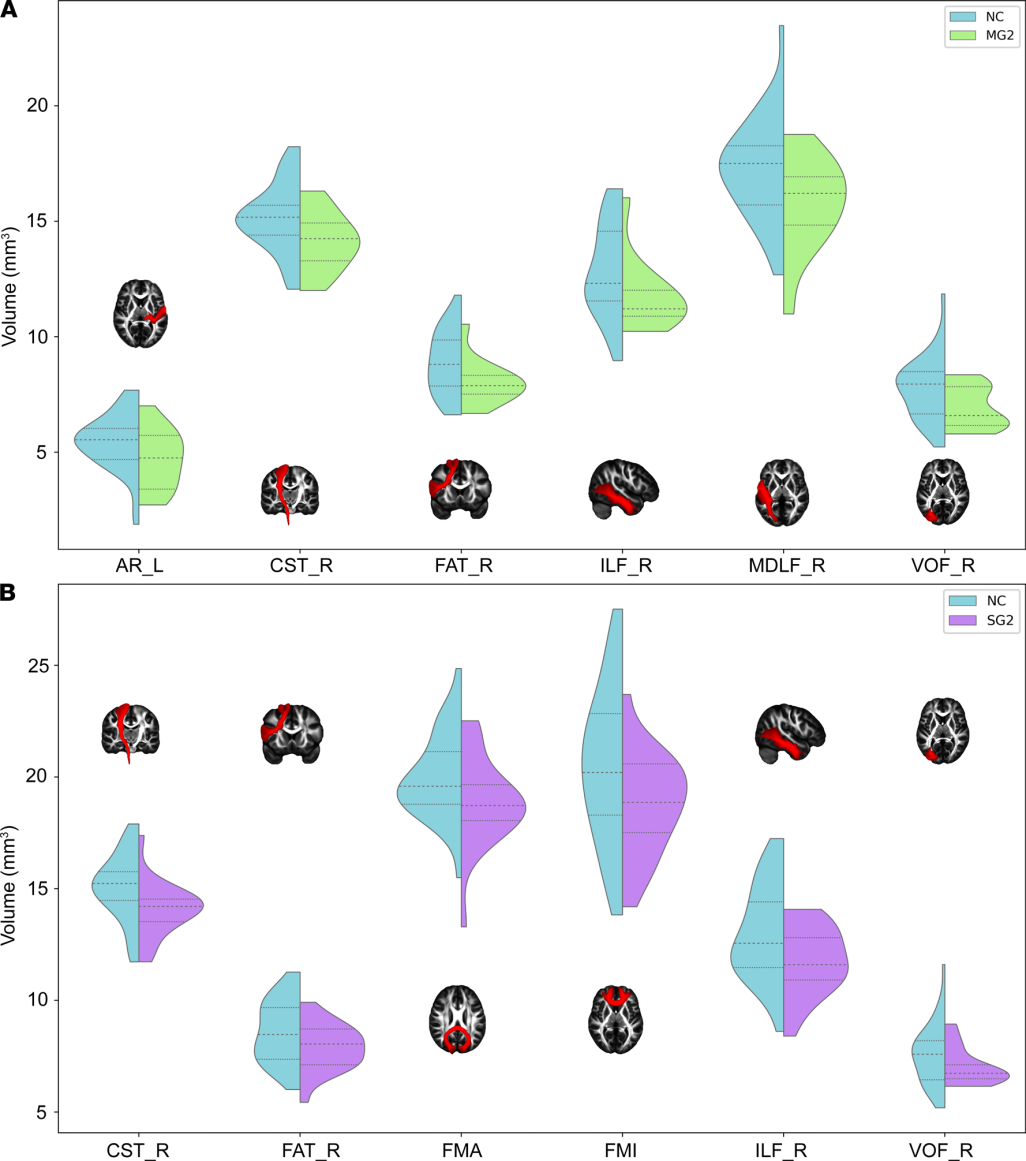
White matter tracts with significant volumetric differences in patients compared with NC. (**A** and **B**) Both MG2 and SG2 showed significantly reduced volumes compared with NC in many white matter tracts. AR, acoustic radiation; CST, corticospinal tract; FAT, frontal aslant tract; FMA, forceps major; FMI, forceps minor; ILF, inferior longitudinal fasciculus; L, left; MDLF, middle longitudinal fasciculus; MG2, mild group at 10 months after discharge; NC, normal control; R, right; SG2, severe group at 10 months after discharge; VOF, vertical occipital fasciculus.

Compared with NC, MG2 showed significantly lower volumes in the left AR (*t* = –1.82, *P* = 0.0414), right CST (*t* = –2.04, *P* = 0.0255), right frontal aslant tract (FAT) (*t* = –1.96, *P* = 0.0292), right inferior longitudinal fasciculus (ILF) (*t* = –1.77, *P* = 0.0395), right middle longitudinal fasciculus (MDLF) (*t* = – 2.08, *P* = 0.0226), and right VOF (*t* = –2.00, *P* = 0.0224) (Figure 7A). Meanwhile, SG2 displayed significantly lower volumes than NC in the right CST (*t* = – 2.52, *P* = 0.0084), right FAT (*t* = –1.99, *P* = 0.0273), forceps major (FMA) (*t* = –2.00, *P* = 0.0253), forceps minor (FMI) (*t* = –2.06, *P* = 0.0233), right ILF (*t* = – 2.30, *P* = 0.0110), and right VOF (*t* = – 1.71, *P* = 0.0449) (Figure 7B).

## Discussion

To our knowledge, this is the first neuroimaging study that explores dynamic brain changes in discharged patients with COVID-19 without clear neurological manifestations at the acute stage. Our findings showed 10-month cross-sectional and longitudinal consequences of COVID-19 on the brain. Overall, cortical thickness differences changed dynamically and finally returned to the baseline. For cortical CBF, the hypoperfusion in SG observed at 3 months tended to recover at 10 months. Subcortical nuclei and white matter differences between groups and within subjects showed various trends, including recoverable and long-term unrecovered differences. After a 10-month recovery period, a decreased nuclei volume in the SG was still much more extensive and profound compared with that in the MG. These cross-sectional and longitudinal brain changes may help clinicians to better understand the potential neuropathogenesis of “long COVID.”

### Cortical differences in comparisons.

There were no significant differences in cortical thickness between NC and patients at 10 months after discharge, both in MG and SG. Only an uptrend in cortical thickness in a few areas was found in the MG2 compared with the MG1. For cortical thickness in SG, different brain regions showed diverse alteration patterns during recovery periods. This may mean that cortical thickness differences changed dynamically and finally returned to the baseline. The diverse course of change may indicate various forms of recovery.

For cortical CBF, there were no significant effects on MG — possibly linked to mild disease status. There was a reduction in hypoperfusion areas in the SG2 than in the SG1 — both compared with the NC. This may indicate that hypoperfusion in some areas has slowly recovered from COVID-19. The hypoperfusion of remaining cortices in SG may implicate potential unrecovered abnormalities from 3 to 10 months after discharge. Furthermore, the peak hypoperfusion value was still observed in the left insula. The insula is hidden under dense arterial and venous blood vessels (15) and is susceptible to pneumonia-induced hypercoagulability, ischemia, and hypoxia, which, as a result, easily leads to obvious hypoperfusion. The hypoperfusion in cortices, especially in the insula, may not only, over time, increase susceptibility to cerebrovascular events, but it may also impair sensory, affective, and cognitive processing (15).

### Subcortical nuclei differences in comparisons.

There were no significant differences in subcortical nuclei volume between MG and NC during any follow-up time periods. Compared with MG1, only the right globus pallidus and left amygdala showed a downward trend in the MG2, implying dynamic but finally recoverable alterations in mild cases. For nuclei volume in SG, atrophy in the left putamen and thalamus observed at 3 months was still found at 10 months, supporting potential unrecovered alterations in these areas. Additionally, newly emerging significant atrophy was observed in the right putamen and accumbens at 10 months after discharge in SG. Compared with the 3-month study, a decreased nuclei volume in the SG was still much more extensive and profound than that in the MG, even after a longer recovery period.

For nuclei CBF in MG, no difference from the NC was reported at the 3-month follow-up. However, compared with the NC, potentially delayed hypoperfusion was noted in some nuclei at 10 months after discharge. For nuclei CBF in SG, significant hypoperfusion in the left caudate, left putamen, and right hippocampus at 3 months after discharge was not observed at 10 months, possibly revealing slow recovery in these nuclei. In addition, compared with the NC, potential unrecovered hypoperfusion was still found in some nuclei in SG at 10 months after discharge.

The high metabolic rate of subcortical nuclei explains the preponderance of subcortical damage in hypoxic-ischemic brain injury and correlates with acute and long-term neurologic syndromes (16, 17). There are spatial relationships among the small arteries and basal nuclei (18). Cerebral small vessel disease is commonly seen in aging and has been associated with subcortical nuclei abnormalities (19). Structural abnormalities in the putamen and thalamus may be potential early markers of major depression (20). Long-term hypoperfusion of subcortical nuclei may also impair the learning of cognitive-motor sequences in response to environmental stimuli (21). Furthermore, the amygdala, accumbens, and hippocampus are putative targets for fear, extinction memory impairment, and emotional dysregulation (22, 23). Potential nuclei abnormalities should be monitored over the long-term to respond to occurrences regarding cerebral small vessel disease, cognitive anomalies, or mental status alterations in many discharged patients with COVID-19 (24).

### White matter differences in comparisons.

A part of the tracts showed significant differences only between the 3-month postdischarge and NC or in the paired tests, without significant differences between the 10-month reexaminations and NC. Although different dynamic patterns were shown, the final results were consistent; they all returned to the baseline. Furthermore, compared with the NC, we found potential unrecovered differences in many tracts during the follow-up, both in MG and SG. Overall, widespread changes were found when different groups were compared. This may indicate that the impact of COVID-19 on white matter is, to a certain extent, sensitive and profound. White matter tracts carry important signals for communicating between different brain areas. Our findings may help increase awareness of a sustained or delayed risk for possible postinfectious neurodegeneration and demyelinating diseases.

### Plasticity-related recovery mechanism.

Regardless of cortices, subcortical nuclei, or white matter tracts, we observed dynamic yet reversible outcomes in some areas both in SG and MG. These may be synthesized to indicate the self-recovery that occurred at various levels of the central nervous system. There were some areas, to varying degrees, that showed a downtrend in the early 3-month stage, presenting with atrophy or hypoperfusion, which may be potentially caused by a natural defense mechanism in order to limit viral replication and signal an adaptive immunological response (25). Meanwhile, there was also an uptrend in some areas indicative of a slight compensatory increase against hypoxic-ischemic injury and inflammation. In order to maintain dynamic equilibrium during recovery, neuroplasticity — which refers to functional and structural alterations in the brain that enable adaptation to changing environments, senescence, or pathological insults (26) — contributes crucially to the reorganization of the brain. Ultimately, it may give rise to shifts from the early confrontation or compensatory stage to the late recovery stage. This previously unexplored plasticity-related recovery may demonstrate a powerful route (27), through which COVID-19 shapes the brain under long-term aggravation.

### Potential mechanism of unrecovered brain abnormalities.

Except for direct virus infection (28), COVID-19 may also adversely affect the brain via multiple indirect pathophysiological pathways driven by systemic inflammation and immune dysregulation (4). Additional inflammation induced by the immune response (29) or hypoxia (30) causes potential neurodegeneration, neuronal loss, and cellular senescence (31). White matter injury is potentially related to ischemia-hypoxia or immunomediated inflammatory demyelination (31). Hypercoagulability (32) related to hypoperfusion may result from endothelial damage, systemic inflammation, and maladaptive immune response (4). Ischemia-hypoxia damage may also result in chronic CBF reduction. Post–SARS-CoV-2 infection causes maladjustment to varying degrees, which may be an important reason for chronic and long-term abnormalities over time. In this study, these long-term potential brain changes may provide objective MRI evidence for prolonged and delayed neurological sequelae reported to date (33, 34).

### Deleterious long-term impact of comorbidities on the brain.

SARS-CoV-2 infects people of all age groups. However, the elderly, especially those aged above 60 years, usually accompanied by comorbidities such as diabetes, cardiovascular diseases, hypertension, and cerebral small vessel disease, are at a higher risk of developing neurological sequelae and have a poorer prognosis (35). Diabetes is related to cognitive impairment, which is often accompanied by abnormal structural and functional MRI of the brain (36). The roles of the immune system and the observed phenomenon of microangiopathy in patients with severe COVID-19 may exacerbate diabetic emergencies and underlying pathophysiology (37). Patients with cardiovascular disease have greater brain atrophy (38) and higher thromboembolism (39). The detrimental effects of cardiovascular disease include acute cerebrovascular disease, cerebral small vessel disease (e.g., microbleeds and white matter hyperintensities in important regions of the brain), and circulatory failure, contributing to early cognitive decline and vascular dementia (40). Among the influences of COVID-19 on cardiovascular and cerebrovascular systems, RAAS is a representative aspect (41). Hypertension-induced brain damage includes cerebral apoplexy, transient ischemic attack, and vascular dementia. Importantly, hypertension appeared consistently as the most prevalent risk factor in patients with COVID-19, presumably related to RAAS (42).

Most current reports have only briefly described the susceptibility of comorbidities to COVID-19, as well as the relationship between comorbidities and increased mortality in patients with COVID-19. The underlying brain changes for these associations are still largely obscure. In this exploratory study, although histories of hypertension, coronal heart disease, and diabetes were regarded as potential confounders and were corrected in our analysis, it will be interesting and valuable to further explore the interactive effects of SARS-CoV-2 and comorbidities on the brain in a large sample.

### Limitations.

There are several limitations in the present study. First, in order to avoid cross-infection, head MRI was not performed at the acute phase, which contributed to a lack of understanding of brain changes from the acute phase to 3 months after discharge. Second, only a small number of patients were willing to participate in the research due to fear of COVID-19 infection and hospitals, and the sample size was inevitably lost again in the longitudinal follow-up. The lack of longitudinal data from healthy controls is another obvious limitation of our study. Additionally, the SG were sicker — having comorbid conditions that are known to affect the brain. A relatively small sample size, imperfect matching, and comparisons of control participants mean that our preliminary findings require further scrutiny, verification, and validation. Next, we acknowledge that the participants lacked professional cognitive measurements, which hinders clinicians from considering some real-world consequences. Finally, this is an exploratory study; even if the nuclei and white matter tract analysis results did not pass the strict multiple comparison correction, a truly uncorrected threshold (*P* < 0.05) was still used to show a significance trend. The future of COVID-19 survivors remains uncertain. We desire a confirmatory study that will be followed by this exploratory study.

### Conclusion.

Cross-sectional and longitudinal follow-up studies characterized dynamic brain changes within 10 months after discharge in patients with COVID-19 without neurological manifestations. In cortices, subcortical nuclei, and white matter tracts of discharged patients, dynamic but recoverable changes coexisted with other long-term potential unrecovered changes. Recovery showed various forms, which revealed a plasticity-related mechanism. However, the remaining potential abnormalities deserve public attention, in order to determine the resolution of risk for “long COVID.” Brain imaging should be considered for long-term monitoring and the assessment of patient recovery, which is critically important for a better understanding of the ongoing neurological effects of COVID-19 and early clinical guidance toward complete recovery.

## Methods

### Participants.

Fifty-one patients who recovered COVID-19 enrolled in our previous 3-month follow-up study (14) were recruited again at 10 months after discharge, and overall, 34 patients revisited our study, including 13 mild type and 21 severe type patients. All patients were recruited from the Department of Infectious Disease in Tongji Hospital of Tongji Medical College of Huazhong University of Science and Technology, Wuhan, China. Detailed inclusion criteria were as follows: (a) patients were 50–70 years old; (b) patients were diagnosed and hospitalized in March 2020; (c) mild- or severe-type COVID-19 was diagnosed according to the World Health Organization interim guidance, without an intensive care unit stay; (d) no clear neurological manifestations were apparent during the acute stage, such as seizures, smell/vision/hearing/taste/memory loss, and impaired mobility; (e) no clear neurological manifestations and no obvious lesion on the conventional MRI were apparent for 10 months after discharge; (f) no history of stroke, head trauma, brain tumors, or epilepsy were apparent. Clinical information and inflammatory markers of patients with COVID-19 were recorded during hospitalizations. Sex-, age-, and education-matched healthy volunteers were enrolled from the community. All participants finished the questionnaires prior to head MRI scan, including handedness, sex, age, education years, underlying diseases, Pittsburgh Sleep Quality Index (PSQI), State-Trait Anxiety Inventory (STAI), and Beck Depression Inventory (BDI).

### MRI scan.

The MRI scan was performed using a 3.0T MR scanner (Discovery MR750, GE Healthcare) with a 32-channel head array coil. The protocol included conventional MRI, structural 3D T1-weighted images (3D-T1WIs), 3D pseudocontinuous arterial spin labeling (3D-pcASL), and high-resolution diffusion tensor imaging (DTI). All scanning parameters, image quality control, and data preprocessing were the same as our previous study (14). More detailed information can be found in our previous study (14). A brief description of preprocessing was provided as follows.

### Cortical and subcortical morphology evaluation.

The T1 images were first denoised by a spatially adaptive filter, and the field inhomogeneity was corrected (43). Then, the T1 images were fed into the morphology evaluation pipeline implemented by Advanced Normalization Tools (ANTs; https://github.com/ANTsX/ANTs; commit ID 6f07ac5) (44). The pipeline was performed for T1 anatomical brain processing using the following steps: (a) brain extraction; (b) brain 7-tissue segmentation (cerebrospinal fluid, gray matter, white matter, ventricle, subcortical nuclei, brain stem, cerebellum); (c) cortical thickness evaluation; (d) registration to MNI template.

### pcASL preprocessing.

According to the kinetic model proposed by Alsop and Detre (45), all raw pcASL images were transferred to the workstation (Advantage Workstation 4.6, GE Medical Systems) to obtain the native CBF map (45). Additionally, we performed a term for the finite labeling duration and corrected the incomplete recovery of the tissue signal (46). The quantitative CBF maps equation was introduced in detail in our previous article (14). Then, all quantitative CBF images were aligned to the T1 images and normalized to the MNI space. The gray matter mask and subcortical nuclei masks obtained from the previous tissue segmentation in brain morphology evaluation were used to extract the cortical and subcortical nuclei CBF, respectively, and the white matter mask was also utilized to avoid contamination between gray matter and white matter (47, 48).

### White matter XTRACT analysis.

The DTI images were preprocessed using the FDT and XTRACT pipeline implemented in FSL (49). Briefly, the approach included the following steps: (a) images were distortion corrected (eddy); (b) diffusion tensors were fitted on the corrected data (dtifit); (c) the probabilistic diffusion model on corrected data was fitted (bedpostx); (d) the b0 map and other maps were all registered to T1 image and MNI template; (e) probabilistic tractography was run on the output of bedpostx (probtrackx); and (f) the XTRACT pipeline was performed to automatically extract a set of carefully dissected tracts in the subject’s native space using probabilistic diffusion tractography. XTRACT is a new, powerful, and reproducible tool, which allows automatic extraction of white matter tracts in the human brain and respects the underlying anatomical variation and individual differences (49). For a given tract, XTRACT (49) produces several postanalysis statistics. Specifically, the volume suggests the most accurate and sensitive statistics in a given tract and is taken as the optimal quantitative statistic. In this study, the final outputs of white matter XTRACT analysis were the volumes of 42 white matter tracts in the MNI space.

### Statistics.

All statistical inferences were conducted using nonparametric permutation tests (5000 random shuffles of subject group labels, *P* < 0.05) by Permutation Analysis of Linear Models (PALM) (50). The multiple comparisons correction of voxel-wise cortical thickness comparisons and CBF comparisons were performed using the TFCE (*P* < 0.05, family-wise error corrected). The statistical multiple comparisons correction of subcortical nuclei volumes, CBFs and white matter tracts volumes was performed by FDR correction (*P* < 0.05). In particular, considering that this is a small sample-sized exploratory study and not a confirmatory study, even if the results did not pass the strict multiple comparisons correction, an uncorrected threshold (*P* < 0.05) was still used to show significant trends.

In addition, demographics and clinical characteristics were compared using a 2-tailed Student’s *t* test, and categorical data were compared using the χ^2^ test. A *P* value less than 0.05 was considered significant. Age, sex, and histories of hypertension, coronal heart disease, and diabetes were regarded as potential confounds and regressed in all group comparisons.

### Study approval.

This human study was approved by the Clinical Institute Ethics Committee of Tongji Hospital, Tongji Medical College, Huazhong University of Science and Technology, and written informed consent was obtained from each participant.

## Author contributions

Concept and design were contributed by YQ, WZ, J Wang, TT, and J Wu. Acquisition, analysis, or interpretation of data were contributed by YQ, WZ, J Wang, TT, J Wu, TC, JL, XP, SY, YZ, and YL. Drafting of the manuscript was contributed by TT, J Wu, J Wang, YQ, WZ, JL, XP, SY, YZ, and YL. Critical revision of the manuscript for important intellectual content was contributed by TC, QN, HX, and FX. Statistical analysis was contributed by J Wang, J Wu, NZ, and AC. Funding acquisition was contributed by WZ, YQ, and J Wu. Administrative, technical, and material support were contributed by TC, NZ, AC, QN, HX, and FX. Supervision was contributed by YQ, WZ, and J Wang. The order of the co–first authors was determined by the extent of their contributions to the work. YQ, WZ, and J Wang have full access to all of the data in the study and take responsibility for the integrity of the data and the accuracy of the data analysis.

## Supplementary Material

Supplemental data

ICMJE disclosure forms

## Figures and Tables

**Table 1:**
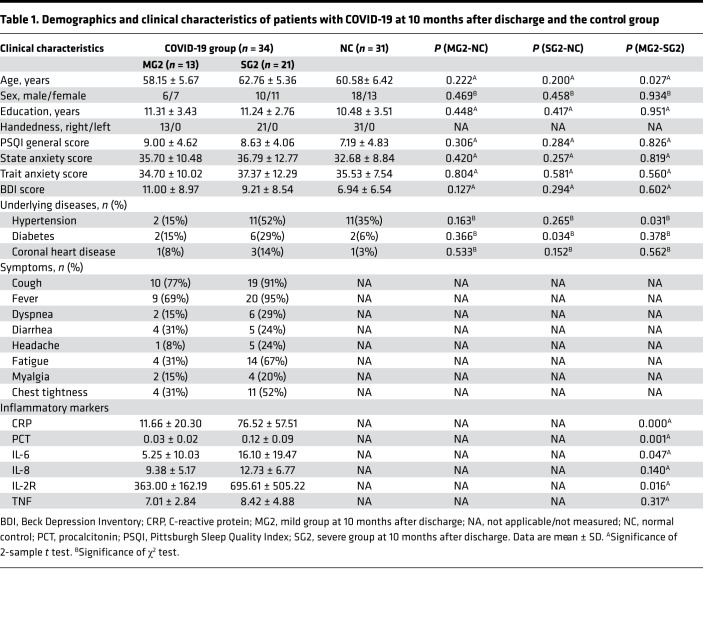
Demographics and clinical characteristics of patients with COVID-19 at 10 months after discharge and the control group

## Version 1 02/22/2022

Electronic publication

## References

[1] Kandemirli SG (2020). Brain MRI findings in patients in the intensive care unit with COVID-19 infection. Radiology.

[2] Li J (2020). Culture versus policy: more global collaboration to effectively combat COVID-19. Innovation (N Y).

[3] Pezzini A, Padovani A (2020). Lifting the mask on neurological manifestations of COVID-19. Nat Rev Neurol.

[4] Iadecola C (2020). Effects of COVID-19 on the nervous system. Cell.

[5] Daugherty SE (2021). Risk of clinical sequelae after the acute phase of SARS-CoV-2 infection: retrospective cohort study. BMJ.

[6] Fotuhi M (2020). Neurobiology of COVID-19. J Alzheimers Dis.

[7] Mao L (2020). Neurologic manifestations of hospitalized patients with coronavirus disease 2019 in Wuhan, China. JAMA Neurol.

[8] Huang C (2021). 6-month consequences of COVID-19 in patients discharged from hospital: a cohort study. Lancet.

[9] Dani M (2021). Autonomic dysfunction in ‘long COVID’: rationale, physiology and management strategies. Clin Med (Lond).

[10] Orrù G (2021). Long-COVID syndrome? A study on the persistence of neurological, psychological and physiological symptoms. Healthcare (Basel).

[11] Petersen MS (2021). Long COVID in the Faroe Islands — a longitudinal study among non-hospitalized patients. Clin Infect Dis.

[12] Vink M, Vink-Niese A (2020). Could cognitive behavioural therapy be an effective treatment for long COVID and post COVID-19 fatigue syndrome? Lessons from the qure study for Q-fever fatigue syndrome. Healthcare (Basel).

[13] Dennis A (2021). Multiorgan impairment in low-risk individuals with post-COVID-19 syndrome: a prospective, community-based study. BMJ Open.

[14] Qin Y (2021). Long-term microstructure and cerebral blood flow changes in patients recovered from COVID-19 without neurological manifestations. J Clin Invest.

[15] Uddin LQ (2017). Structure and function of the human insula. J Clin Neurophysiol.

[16] Kulick-Soper CV (2020). Pearls & Oy-sters: bilateral globus pallidus lesions in a patient with COVID-19. Neurology.

[17] Pasternak JF, Gorey MT (1998). The syndrome of acute near-total intrauterine asphyxia in the term infant. Pediatr Neurol.

[18] Wei N (2021). A processing pipeline for quantifying lenticulostriate artery vascular volume in subcortical nuclei. Front Neurol.

[19] Liem MK (2012). 7 T MRI reveals diffuse iron deposition in putamen and caudate nucleus in CADASIL. J Neurol Neurosurg Psychiatry.

[20] Lu Y (2016). The volumetric and shape changes of the putamen and thalamus in first episode, untreated major depressive disorder. Neuroimage Clin.

[21] Fino E (2009). Asymmetric spike-timing dependent plasticity of striatal nitric oxide-synthase interneurons. Neuroscience.

[22] Kumari P (2020). Hypobaric hypoxia induced fear and extinction memory impairment and effect of Ginkgo biloba in its amelioration: Behavioral, neurochemical and molecular correlates. Behav Brain Res.

[23] Pessoa L (2017). A network model of the emotional brain. Trends Cogn Sci.

[24] Newcombe VFJ (2021). Neuroanatomical substrates of generalized brain dysfunction in COVID-19. Intensive Care Med.

[25] Baz-Martinez M (2016). Cell senescence is an antiviral defense mechanism. Sci Rep.

[26] von Bernhardi R (2017). What is neural plasticity?. Adv Exp Med Biol.

[27] Sampaio-Baptista C, Johansen-Berg H (2017). White matter plasticity in the adult brain. Neuron.

[28] Matschke J (2020). Neuropathology of patients with COVID-19 in Germany: a post-mortem case series. Lancet Neurol.

[29] Huang C (2020). Clinical features of patients infected with 2019 novel coronavirus in Wuhan, China. Lancet.

[30] Kantonen J (2020). Neuropathologic features of four autopsied COVID-19 patients. Brain Pathol.

[31] Hascup ER, Hascup KN (2020). Does SARS-CoV-2 infection cause chronic neurological complications?. Geroscience.

[32] Helms J (2020). High risk of thrombosis in patients with severe SARS-CoV-2 infection: a multicenter prospective cohort study. Intensive Care Med.

[33] Raahimi MM (2021). Late onset of Guillain-Barre syndrome following SARS-CoV-2 infection: part of ‘long COVID-19 syndrome’?. BMJ Case Rep.

[34] Del Brutto OH (2021). Cognitive decline among individuals with history of mild symptomatic SARS-CoV-2 infection: a longitudinal prospective study nested to a population cohort. Eur J Neurol.

[35] Ejaz H (2020). COVID-19 and comorbidities: deleterious impact on infected patients. J Infect Public Health.

[36] Zilliox LA (2016). Diabetes and cognitive impairment. Curr Diab Rep.

[37] Muniangi-Muhitu H (2020). Covid-19 and diabetes: a complex bidirectional relationship. Front Endocrinol (Lausanne).

[38] van der Veen PH (2014). Brain volumes and risk of cardiovascular events and mortality. The SMART-MR study. Neurobiol Aging.

[39] Gasmi A (2021). Interrelations between COVID-19 and other disorders. Clin Immunol.

[40] de Roos A (2017). Magnetic resonance imaging of cardiovascular function and the brain: is dementia a cardiovascular-driven disease?. Circulation.

[41] Kurz DJ, Eberli FR (2020). Cardiovascular aspects of COVID-19. Swiss Med Wkly.

[42] Tadic M (2020). COVID-19 and arterial hypertension: hypothesis or evidence?. J Clin Hypertens (Greenwich).

[43] Manjon JV, Coupe P (2016). volBrain: An online MRI brain volumetry system. Front Neuroinform.

[44] Tustison NJ (2014). Large-scale evaluation of ANTs and FreeSurfer cortical thickness measurements. Neuroimage.

[45] Alsop DC, Detre JA (1996). Reduced transit-time sensitivity in noninvasive magnetic resonance imaging of human cerebral blood flow. J Cereb Blood Flow Metab.

[46] Wu B (2014). Intra- and interscanner reliability and reproducibility of 3D whole-brain pseudo-continuous arterial spin-labeling MR perfusion at 3T. J Magn Reson Imaging.

[47] van Gelderen P (2008). Pittfalls of MRI measurement of white matter perfusion based on arterial spin labeling. Magn Reson Med.

[48] Xu G (2010). Reliability and precision of pseudo-continuous arterial spin labeling perfusion MRI on 3.0 T and comparison with 15O-water PET in elderly subjects at risk for Alzheimer’s disease. NMR Biomed.

[49] Warrington S (2020). XTRACT — standardised protocols for automated tractography in the human and macaque brain. Neuroimage.

[50] Winkler AM (2014). Permutation inference for the general linear model. Neuroimage.

