# Supplementary material for: Long-term follow-up of dynamic brain changes in patients recovered from COVID-19 without neurological manifestations
Source: JCI Insight. 2022 Feb 22;7(4):e155827. doi: 10.1172/jci.insight.155827 (PMC8876627; doi:10.1172/jci.insight.155827)
Supplement: Supplemental data [file jciinsight-7-155827-s145.pdf]

Cortical thickness comparisons

Compared with MG1, MG2 exhibited thicker gray matter in the left limbic areas (MNI: -35 -27 -16, t: 8.37), right medial frontal gyrus (MNI: 11 37 -23, t: 8.77), left inferior frontal cortex (MNI: -13 37 -24, t: 5.86), right parahippocampal gyrus (MNI: 32 -39 -23, t: 7.18), left superior frontal gyrus (MNI: -10 55 -21, t: 4.74), left middle frontal gyrus (MNI: -29 38 -9, t: 8.74), left medial frontal gyrus (MNI: -3 40 -10, t: 7.20), right middle frontal gyrus (MNI: 26 53 4, t: 8.48), left medial orbital-frontal cortex (MNI: -4 59 -7, t: 6.93), left temporal-parietal cortex (MNI: -43 -64 14, t: 6.07), and left medial frontal gyrus (MNI: -8 52 15, t: 7.49). Meanwhile, compared with SG1, SG2 displayed not only gray matter atrophy in the right precentral and postcentral gyrus (MNI: 50 -19 47, t: -6.67), right superior temporal gyrus (MNI: 43 -1 -18, t: -5.46), right inferior parietal lobule (MNI: 60 -35 31, t: -5.49), and right supplementary motor area (MNI: 5 7 59, t: -6.08), but also thicker gray matter in the left limbic areas (MNI: -32 -46 -7 , t: 8.48), left inferior temporal gyrus (MNI: -55 -12 -31, t: 6.15), left middle frontal gyrus (MNI: -31 58 8, t: 6.50), and left superior frontal gyrus (MNI: -25 52 16, t: 9.01). The provided MNI coordinates were the peaks of areas.
